# The neutrophil-to-lymphocyte ratio is associated with mild cognitive impairment in community-dwelling older women aged over 70 years: a population-based cross-sectional study

**DOI:** 10.3389/fnagi.2023.1261026

**Published:** 2023-09-14

**Authors:** Shengjie Li, Xiaoyu Chen, Mengze Gao, Xingyu Zhang, Peipei Han, Liou Cao, Jing Gao, Qiongying Tao, Jiayi Zhai, Dongyu Liang, Qi Guo

**Affiliations:** ^1^Department of Rehabilitation Medicine, Shanghai University of Medicine and Health Sciences Affiliated Zhoupu Hospital, Shanghai, China; ^2^School of Sports and Health, Tianjin University of Sport, Tianjin, China; ^3^Department of Nephrology, Molecular Cell Lab for Kidney Disease, Ren Ji Hospital, Shanghai Jiao Tong University School of Medicine, Shanghai, China; ^4^General Practice Clinic, Pujiang Community Health Service Center in Minhang District, Shanghai, China; ^5^Jiading Subdistrict Community Health Center, Shanghai, China; ^6^Clinical Research Center, Jiading District Central Hospital Affiliated Shanghai University of Medicine and Health Sciences, Shanghai, China

**Keywords:** inflammations, mild cognitive impairment, sex difference, population-based study, neutrophil-to-lymphocyte ratio (NLR)

## Abstract

**Background:**

The neutrophil-to-lymphocyte ratio (NLR) is a marker of inflammation that can be obtained quickly, conveniently, and cheaply from blood samples. However, there is no research to explore the effects of sex and age on the relationship between the NLR and mild cognitive impairment (MCI) in community-dwelling older adults.

**Methods:**

A total of 3,169 individuals aged over 60 years in Shanghai were recruited for face-to-face interviews, and blood samples were collected. MCI was assessed by the Mini-Mental State Examination (MMSE) and the Instrumental Activities of Daily Living (IADL) scale, and neutrophil count and lymphocyte counts were measured in fasting blood samples. The NLR was calculated by dividing the absolute neutrophil count by the absolute lymphocyte count.

**Results:**

In females, the NLR in the MCI group was significantly higher than that in the cognitively normal group (2.13 ± 0.94 vs. 1.85 ± 0.83, *p* < 0.001) but not in men. Logistic regression showed that a higher NLR was an independent risk factor for MCI in women [odds ratio (OR) = 1.28; 95% confidence interval (CI) = 1.09–1.49]. In addition, the elevated NLR quartile was associated with an increased risk of MCI, especially in women older than 70 years (p-value for trend = 0.011).

**Conclusion:**

Compared with males, female MCI patients had a significantly higher NLR than cognitively normal controls. In addition, elevated NLR was found to be significantly associated with MCI risk in women older than 70 years. Therefore, elderly Chinese women with a higher NLR value may be the target population for effective prevention of MCI.

## Introduction

With the rise in the aging of the population, age-related cognitive decline has become a major global public health concern and caused a huge economic and social burden, particularly in developing countries (Winblad et al., 2004). Mild cognitive impairment (MCI) is considered a transitional state between normal cognitive function and dementia. MCI is characterized as a risk factor for dementia (Liu et al., 2022). Patients with MCI will progress to dementia at a significantly greater rate of acceleration than that of healthy people of a similar age. Approximately 32% of patients with MCI develop Alzheimer’s disease (AD) after five years (2021 Alzheimer’s disease facts and figures, 2021). However, guidelines suggest that early detection and timely treatment of MCI can reverse the decline in cognitive function (Petersen et al., 2018). It is possible to prevent or delay the onset of dementia or AD with early diagnosis of and intervention in MCI.

The immune system is irreversibly impaired by aging, and that impairment is considered to be the most important risk factor for MCI. Inflammatory processes play a complex role in the progression of cognitive impairment (Pillai et al., 2019). Disorders of immune and inflammatory responses have been considered important risk components of MCI. Previous literature suggests that many common peripheral blood parameters may be novel inflammatory markers and may be associated with the pathogenesis or prognosis of central nervous system diseases (Shen et al., 2019). According to several studies, abnormal levels of peripheral inflammatory markers have been widely associated with MCI (Sağlam Aykut et al., 2018; Dong et al., 2019; An et al., 2020). According to a study from China, an increased risk of MCI was significantly associated with an elevated neutrophil-to-lymphocyte ratio (NLR) (An et al., 2020). Another study showed that the NLR and neutrophil percentage (neutrophil%) may be useful for identifying patients with MCI, as biomarkers in routine blood samples may correlate with cognitive impairment (Dong et al., 2019). However, there was also a study that showed a negative correlation between cognitive function and the NLR (Sağlam Aykut et al., 2018). Further, these studies were limited by relatively small sample sizes and did not consider several important confounding factors, especially sex and age.

In fact, age and sex may affect the relationship between MCI and NLR. A study from South America showed that the NLR had different age distribution characteristics between sexes. Many studies have demonstrated that sex hormones are closely linked to inflammation and cognitive impairment (Mf et al., 2016; Boccardi et al., 2019). Compared with men, postmenopausal women have a more rapid decline in peripheral sex hormone levels, as well as a higher inflammatory burden (Engler et al., 2016). According to a study by Minhas et al. (2021), aging promotes maladaptive inflammation that affects cognitive function. A decline in cognitive function as well as an increased risk of dementia is associated with aging and premature menopause in women (Duarte et al., 2016). The relationship between MCI and peripheral inflammatory markers in different sexes and ages remains uncertain. To date, no study has investigated the relationship between the NLR and MCI when stratified by age and sex, so the effect of age and sex on this relationship remains unclear.

Therefore, the aim of this study was to explore the relationship between MCI and peripheral inflammatory markers in different sex and age groups and provide a theoretical basis for the prevention and identification of MCI through an increased understanding of MCI.

## Methods

### Subjects

All participants, who were residents from six communities in Shanghai, were invited to engage in a comprehensive geriatric assessment in Shanghai from 2019 to 2023, during which they completed a face-to-face interview questionnaire as well as a physical examination. The study was approved by the Ethics Committee of Shanghai University of Medicine and Health Sciences, and the methods were carried out in accordance with the principles of the Declaration of Helsinki.

The inclusion criteria for this study were age 60 years or older and completion of relevant tests. The exclusion criteria were as follows: (1) persons with severe mental disorders, dementia, or other neurodegenerative diseases; (2) persons who did not provide informed consent or were unable to communicate with investigators; (3) persons with severe sensory impairments and unable to complete the assessment; and (4) persons for whom blood samples were not collected. A total of 4,046 participants were enrolled in the study. Of these, 310 individuals had an incomplete assessment of cognition and were excluded from the analysis. Additionally, 544 participants lacked blood samples, and 23 lacked other covariates and were also excluded from the analysis. The final analysis of the study included 3,169 participants (1,368 men and 1,801 women).

### Definition of MCI

MCI was defined as patients with low cognitive ability [Mini-Mental State Examination (MMSE) score below the educational level cut point] and normal activities of daily living [Instrumental Activities of Daily Living (IADL) score ≥ 6] (Arevalo-Rodriguez et al., 2015). The Chinese version of the MMSE was used to assess cognitive impairment, with the following cutoff points: illiterate ≤17 points, primary school ≤20 points, and high school education level ≤ 24 points (Liu et al., 2022). The MMSE has been shown to be a reliable indicator of the cognitive status of Chinese individuals (Zhang et al., 2006).

**Figure 1:**
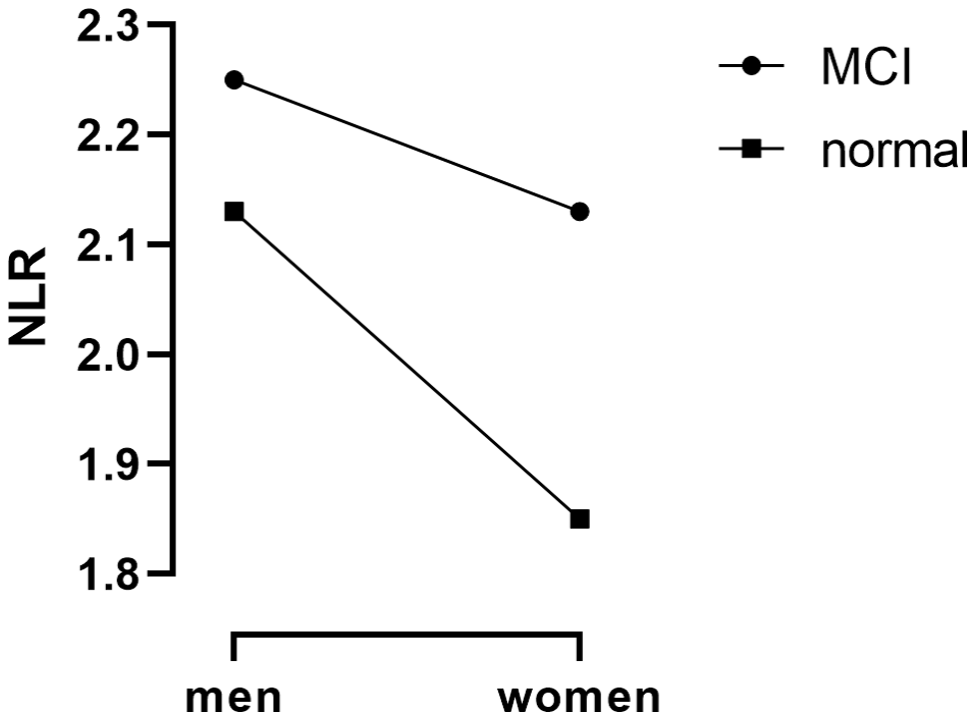
The relationship between NLR and MCI in men and women. Data are shown as medians.

**Figure 2:**
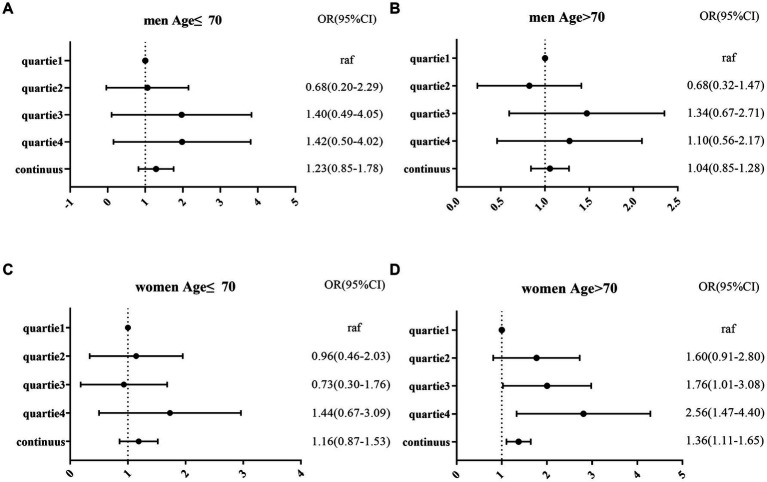
Logistic regression of MCI and NLR after adjusted model in four subgroups by sex and age (A–D).

### Inflammatory markers

Peripheral inflammatory cells, including white blood cell (WBC), neutrophil, lymphocyte, and monocyte counts, were assessed by a hematology analyzer XE-2100 (Sysmex). NLR values were calculated by dividing the absolute neutrophil count by the absolute lymphocyte count: neutrophil count/lymphocyte count.

### Covariates

All participants were invited to participate in a face-to-face interview to answer a standardized questionnaire to obtain baseline data. Baseline data on sociodemographic characteristics, chronic disease status, and health behaviors were treated as covariates. Demographic characteristics included age, sex, height, weight, and education level. Health behaviors included smoking and alcohol consumption habits. Depressive symptoms were assessed using the Chinese version of the Geriatric Depression Scale (GDS), which was standardized in 1996 after its reliability and validity were tested (Yesavage et al., 1982). Nutritional status was measured by the Mini Nutritional Assessment (MNA) containing 18 items. A score of more than 23.5 was well-nourished, a score of 17 to 23.5 was at risk of malnutrition, and a score of less than 17 was malnourished. Physical activity was assessed by the International Physical Activity Questionnaire (IPAQ), total minutes of vigorous activity, moderate activity, and walking during a week were multiplied by 8.0, 4.0, and 3.3, respectively, and then added to represent total physical activity. The medical history of the participants was based on a combination of questionnaires answered by the participants, physician diagnoses, and taking medications or other current or past treatments, including hypertension, hyperlipidemia, diabetes mellitus, or coronary heart disease. Fasting plasma glucose was used to diagnose diabetes, lipid markers was used to diagnose hyperlipidemia, blood pressure was used to diagnose hypertension.

### Statistical analysis

The following data analysis was conducted to investigate the correlation between MCI and inflammation among different sexes. Sociodemographic and health-related characteristics were collected at baseline. Normally distributed data are expressed as the mean and standard deviation, while nonnormally distributed data are expressed as the median and quartile. The t test or Mann–Whitney U test was used to analyze differences in baseline characteristics. Categorical variables were expressed as percentages (%) and analyzed using the χ2 test. Logistic regression models were used to examine the relationship between MCI and peripheral inflammatory cells by sex. Adjusted model included age, BMI, education, depression, widow, hypertension, coronary heart disease, smoking, and drinking. Further, we explored the relationship between MCI and age and sex using logistic regression. Adjusted model included BMI, education, depression, widow, hypertension, coronary heart disease, smoking, and drinking. The results are expressed as odds ratios, 95% confidence intervals, and corresponding *p* values. *p* value for trend was calculated from a one degree-of-freedom trend test. A *p* value of less than 0.05 was considered to indicate statistical significance. Statistical analysis was performed using SPSS v26.0.

## Results

In total, 3,169 individuals participated in the study (mean age, 72.18 ± 5.34 years; 56.8% women). The prevalence of MCI was 8.4% and 11.5% among men and women, respectively. Table 1 presents the characteristics of the study participants (*n* = 3,169) stratified by sex. In men, participants with MCI tended to have a lower level of nutritional status and education but had high levels of depression and hypertension (*p* < 0.05). In women, participants with MCI tended to have a lower level of education, lymphocytes% and lymphocytes but had a higher level of age, neutrophils%, NLR, hyperlipidemia and platelets; in addition, they were more likely to be widows (*p* < 0.05) (Figure 1).

**Table 1:** Baseline characteristics of study participants with and without mild cognitive impairment.

Characteristic	Men			Women		
	Normal (*n* = 1,252)	MCI (*n* = 116)	*p* value	Normal (*n* = 1,593)	MCI (*n* = 208)	*p* value
Age (years)	72.37 ± 5.20	74.19 ± 6.53	0.004	71.56 ± 5.10	74.60 ± 6.11	<0.001
BMI (kg/m^2^)	23.84 ± 3.17	23.88 ± 3.51	0.900	23.81 ± 3.48	24.20 ± 3.52	0.166
Widow (%)	92 (7.3)	14 (12.1)	0.069	341 (21.4)	65 (31.3)	0.001
Education (%)			0.001			<0.001
Illiteracy	37 (3.0)	11 (9.5)		183 (11.5)	80 (38.5)	
Primary school	447 (35.7)	39 (33.6)		604 (37.9)	78 (37.5)	
Junior high school or above	768 (61.3)	66 (56.9)		806 (50.6)	50 (24.0)	
Drinking (%)	523 (41.8)	51 (44.0)	0.647	146 (9.2)	15 (7.2)	0.353
Smoking (%)	389 (31.1)	44 (37.9)	0.129	10 (0.6)	0 (0)	0.252
MNA	19.47 ± 11.25	22.50 ± 8.11	0.007	21.88 ± 8.83	20.36 ± 9.45	0.304
IPAQ (Met-min/wk)	5,175 (1431–6,516)	4,591 (938–5,219)	0.810	4,270 (1509–8,106)	3,822 (1386–7,413)	0.090
Chronic disease (%)						
Hyperlipidemia	357 (28.5)	37 (31.25)	0.442	670 (42.1)	69 (33.2)	0.001
Hypertension	778 (62.1)	83 (71.6)	0.045	1,003 (63.0)	148 (71.2)	0.021
Diabetes	259 (20.7)	28 (24.1)	0.382	306 (19.2)	47 (22.6)	0.247
Coronary heart disease	261 (21.6)	30 (25.9)	0.294	431 (27.1)	74 (35.6)	0.010
Depression (%)	105 (8.4)	23 (19.8)	<0.001	225 (14.1)	43 (20.7)	0.013
Peripheral Blood Biomarkers						
WBC (×10^9^/ L)	6.21 ± 1.53	6.08 ± 1.40	0.410	5.86 ± 1.45	5.88 ± 1.46	0.854
Lymphocytes%	31.93 ± 8.38	30.72 ± 8.13	0.135	34.89 ± 8.29	32.43 ± 8.34	<0.001
Neutrophils%	60.44 ± 9.57	62.05 ± 9.63	0.082	58.48 ± 9.22	61.89 ± 9.25	<0.001
Lymphocytes (×10^9^/L)	1.96 ± 0.66	1.85 ± 0.60	0.083	2.02 ± 0.63	1.87 ± 0.57	0.001
Neutrophils (×10^9^/L)	3.77 ± 1.21	3.80 ± 1.11	0.837	3.46 ± 1.14	3.68 ± 1.20	0.009
NLR	2.13 ± 1.01	2.25 ± 0.96	0.177	1.85 ± 0.83	2.13 ± 0.94	<0.001

MCI, mild cognitive impairment; BMI, body mass index; MNA, Mini-nutritional assessment; IPAQ, international physical activity questionnaire; WBC, white blood cell; NLR, neutrophil-to-lymphocyte ratio.

Table 2 presents the results of the sex-specific crude and adjusted associations between inflammatory markers and the prevalence of MCI. Among women, the NLR was positively associated with the presence of MCI in all models. The prevalence of MCI was highest in the fourth quartile of the NLR [odds ratio (OR) = 2.10; 95% confidence interval (CI) = 1.35–3.25]. However, in men, no significant association of these peripheral inflammatory parameters with MCI was found in the final multivariate model. Similar results were obtained when these peripheral inflammatory parameters were analyzed as continuous variables.

**Table 2:** Association between NLR and MCI in older adults by sex.

NLR	Men		Women	
	Crude	Adjusted model	Crude	Adjusted model
Quartile 1	1.00 (ref)	1.00 (ref)	1.00 (ref)	1.00 (ref)
Quartile 2	0.68 (0.36–1.28)	0.66 (0.35–1.26)	1.47 (0.96–2.25)	1.34 (0.86–2.08)
Quartile 3	1.37 (0.78–2.38)	1.31 (0.74–2.33)	1.68 (1.09–2.60)	1.38 (0.88–2.18)
Quartile 4	1.26 (0.73–2.17)	1.16 (0.66–2.04)	2.61^***^ (1.72–3.96)	2.10^**^ (1.35–3.25)
Continuous	1.12 (0.94–1.33)	1.08 (0.91–1.30)	1.38^***^ (1.19–1.59)	1.28^**^ (1.09–1.49)

Adjusted model: adjusted for age, education, BMI, depression, widow, hypertension, coronary heart disease, smoking, and drinking. *p < 0.05; **p < 0.01; ***p < 0.001 Values are odds ratio (95% confidence interval). BMI, body mass index; MCI, mild cognitive impairment; NLR, neutrophil-to-lymphocyte ratio.

Table 3 shows that the prevalence of MCI was higher in the third (OR = 1.76; 95% CI = 1.01–3.08), and fourth quartiles of the NLR (OR = 2.56; 95% CI = 1.47–4.40) than in the first quartile of women older than 70 years. This suggests that the risk of MCI increased with increasing NLR in older women (*p* value for trend = 0.011) but not in younger women and men (See Figure 2).

**Table 3:** Association between NLR and MCI in four subgroups by sex and age.

NLR	Men				Women			
	Age ≤ 70		Age > 70		Age ≤ 70		Age > 70	
	Crude	Adjusted model	Crude	Adjusted model	Crude	Adjusted model	Crude	Adjusted model
Quartile 1	1.00 (ref)	1.00 (ref)	1.00 (ref)	1.00 (ref)	1.00 (ref)	1.00 (ref)	1.00 (ref)	1.00 (ref)
Quartile 2	0.62 (0.19–2.00)	0.68 (0.20–2.29)	0.70 (0.89–1.33)	0.68 (0.32–1.47)	0.96 (0.46–2.00)	0.96 (0.46–2.03)	1.81^*^ (1.06–3.09)	1.60 (0.91–2.80)
Quartile 3	1.39 (0.52–3.74)	1.40 (0.49–4.05)	1.34 (0.67–3.47)	1.34 (0.67–2.71)	0.82 (0.34–1.95)	0.73 (0.30–1.76)	2.09^*^ (1.22–3.55)	1.76^*^ (1.01–3.08)
Quartile 4	1.32 (0.49–3.53)	1.42 (0.50–4.02)	1.22 (0.63–2.34)	1.10 (0.56–2.17)	1.74 (0.84–3.62)	1.44 (0.67–3.09)	3.04^***^ (1.81–5.11)	2.56^**^ (1.47–4.40)
P value for trend	0.381	0.285	0.443	0.611	0.239	0.395	0.011	0.011
Continuous	1.19 (0.85–1.68)	1.23 (0.85–1.78)	1.09 (0.89–1.33)	1.04 (0.85–1.28)	1.24 (0.96–1.61)	1.16 (0.87–1.53)	1.43^***^ (1.19–1.72)	1.36^**^ (1.11–1.65)

Adjusted model: adjusted for BMI, education, depression, widow, hypertension, coronary heart disease, smoking, and drinking. *p < 0.05; **p < 0.01; ***p < 0.001 values are odds ratio (95% confidence interval). BMI, body mass index; MCI, mild cognitive impairment; NLR, neutrophil-to-lymphocyte ratio.

## Discussion

In this cross-sectional study, we found that the prevalence of MCI differed significantly between men and women and was influenced by age. We evaluated the effect of sex and age on the relationship between MCI and peripheral inflammatory markers in elderly individuals in rural areas of China. We found significant differences in NLR, lymphocytes and neutrophils% between patients with MCI and those without MCI in women but not in men. In addition, the NLR was significantly associated with the prevalence of MCI in women over 70 years of age but not in the other groups.

Studies in China have shown a higher risk of MCI in older women than in men (Nie et al., 2011; Xue et al., 2018), but studies from other countries have not found sex-based differences in MCI prevalence (Ravaglia et al., 2008; Katz et al., 2012). Additionally, some studies have shown a higher prevalence of MCI in men than in women (Bae et al., 2015). Sex differences in MCI prevalence may be due to risk factors like hyperlipidemia and malnutrition, along with a lower education level among Chinese elderly women. These risk factors were also found in our study. Inadequate education for women may lead to poorer career achievement, lower earnings, poorer health, and lower cognitive outcomes compared to men. In addition, the present study found that the association between hyperlipidemia and MCI was seen only in women, and women with hyperlipidemia had higher cognitive function than women without hyperlipidemia. This finding is consistent with a previous study conducted by our team (Liu et al., 2022). Women with MCI were found to have a protective factor against hyperlipidemia (Kim and Park, 2017). Studies indicate that higher serum cholesterol levels are associated with a reduced risk of cognitive impairment. However, the mechanisms linking sex, hyperlipidemia, and cognitive impairment are not well understood. Similar to the present study, a recent meta-analysis has shown sex differences in the relationship between cognitive impairment and malnutrition. Cognitive impairment was an independent predictor of malnutrition development at the 2-year follow-up among female participants (Corish and Bardon, 2019). Further research is required to investigate the underlying mechanisms.

Chronic low-grade systemic inflammation is regarded as one of the most relevant features characterizing aging and related diseases. Studies have shown that NLR values are significantly higher in AD (Kuyumcu et al., 2012) and MCI (An et al., 2020) patients than in controls. This is consistent with the results of our study, there is a significant difference in NLR between women with and without MCI (2.13 ± 0.94 vs. 1.85 ± 0.83). It has also been suggested that the NLR is a predictor of cognitive impairment (Halazun et al., 2014). Dong et al. (2019) showed that the NLR, neutrophil%, and mean platelet volume (MPV) were potentially useful for the identification of patients with MCI. Le et al. showed that peripheral B lymphocyte depletion was associated with increased Aβ burden (le Page et al., 2017). In contrast, a case–control study by Sağlam Aykut et al. (2018) reported a negative correlation between cognitive function and the NLR. In addition, other studies have shown that NLR values were significantly lower in Parkinson’s disease patients with MCI compared with those without MCI (Contaldi et al., 2022). The differences in results may be due to differences in sample size, age, disease, and scales that measure cognitive function. Moreover, NLR values may vary among individuals from different racial and ethnic backgrounds. Nonetheless, several studies and this study have demonstrated a correlation between the NLR and cognitive impairment, suggesting that peripheral inflammation may play a crucial role in the pathogenesis of MCI.

Sex differences in immune systems can affect memory and cognitive function (Zhu et al., 2021). Women generally have stronger immune responses than men, which can lead to higher susceptibility to infections in men and a higher incidence of autoimmune disorders in women (Tronson and Collette, 2017). The APOE4 allele, which is the greatest genetic risk factor for cognitive impairment, has been shown to increase susceptibility to inflammation (Zhu et al., 2021). The APOE4 allele increases the risk of cognitive impairment to a significantly greater extent in women than in men (Altmann et al., 2014). Moreover, sex hormones can cause differences in the biology and/or effector functions of neutrophils between men and women (Bouman et al., 2005). Estradiol and progesterone, two types of female hormones, have been found to delay the death of neutrophils and change their movement (Jaillon et al., 2019). Older women exhibit different inflammatory responses under the influence of multiple pathways, including an increase in neutrophils and a decrease in lymphocytes (Dodd and Menon, 2022). Meanwhile, sex differences in age-related reduction of sex hormones may contribute to the difference in cognitive impairment between men and women (Li and Singh, 2014). Sex hormones and the differences in related genes such as APOE4 allele, can all be associated with maladaptive microglia as well as sex differences in the immune system (Mf et al., 2016). Coronary heart disease is associated with APOE4 allele (Liu et al., 2021), and we found its incidence is significantly higher in women than in men (women,28.0% vs. men, 22.0%). Sex-specific differences in microglial responses to neuroinflammation may imply that women are more susceptible to inflammatory stimuli leading to cognitive decline, whereas men are more tolerant to these stimuli (Zhu et al., 2021). Therefore, sex differences may be an important factor affecting inflammation and cognitive function. Further research is needed to understand the mechanisms underlying these sex-specific differences.

The aging of peripheral cells may lead to chronic systemic inflammation during the aging process and the excessive release of various inflammatory mediators under inflammatory stimulation (Budamagunta et al., 2023). Animal models suggest that aging may reduce neutrophil clearance, which could compromise inflammation resolution following injury (Ramos-Cejudo et al., 2021). Systemic inflammation can promote aging-like brain changes, which may lead to a trajectory of cognitive decline. In addition, aging can affect cholinergic regulation of the nervous system and reduce acetylcholine activity, thereby activating multiple signaling pathways that regulate oxidative stress, inflammation of the nervous system, and interference with brain neurotrophic factors (Wang et al., 2023). Therefore, in age-related and pathological conditions, the accumulation of cell aging in peripheral tissues may lead to impaired memory function. A cohort study identified age as an important factor affecting cognitive ability in rural elderly people and related to the progression of cognitive impairment (Xu et al., 2023). Our study also observed that MCI patients were older than controls in both men and women. Aging increases the risk of cognitive impairment and causes low-grade systemic inflammation in the peripheral and central areas, but the interaction among these three factors is unclear and requires further study.

This study presents several strengths. First, this study sampled a relatively large number of settled older men and women who were relatively stable in their communities for a long period of time. Second, the study subjects came from different communities, and they had different lifestyles, which made our study subjects more representative. However, there are several limitations in this study. First, the participants in this study were in relatively good health, as we excluded patients who could not complete a full medical evaluation. Second, cognitive function was assessed by the MMSE. Therefore, this study could not further diagnose different types of cognitive impairment, including non-amnestic MCI and amnestic MCI. Third, alternatively, this study did not consider other factors that may contribute to cognitive impairment, including the APOE4 allele, atrial fibrillation, and carotid stenosis. Finally, this study was a cross-sectional study, and further longitudinal studies are needed to determine the causal relationship between MCI and inflammation.

## Conclusion

In conclusion, we found that the NLR was significantly higher in female patients with MCI than in cognitively normal controls but it was not significantly increased in men. Moreover, the NLR was significantly associated with the risk of MCI in women older than 70 years of age. Further prospective studies are needed to confirm the causal relationship between inflammation and MCI in elderly women. This study has important implications for the early differential diagnosis of MCI and the improvement of cognitive status in elderly individuals.

## Data availability statement

The raw data supporting the conclusions of this article will be made available by the authors, without undue reservation.

## Ethics statement

The studies involving humans were approved by the Institutional Review Board of the Ethics Committee at Shanghai University of Medicine and Health Sciences. The studies were conducted in accordance with the local legislation and institutional requirements. Written informed consent for participation in this study was provided by the participants' legal guardians/next of kin.

## Author contributions

SL: Formal analysis, Investigation, Methodology, Writing – original draft, Funding acquisition. XC: Conceptualization, Data curation, Writing – review & editing. MG: Investigation, Methodology, Project administration, Writing – review & editing. XZ: Data curation, Investigation, Project administration, Writing – review & editing. PH: Investigation, Writing – review & editing. LC: Project administration, Supervision, Writing – review & editing. JG: Resources, Writing – review & editing. QT: Resources, Writing – review & editing. JZ: Writing – review & editing. DL: Writing – review & editing. QG: Formal analysis, Supervision, Writing – review & editing.

## Funding

The author(s) declare financial support was received for the research, authorship, and/or publication of this article. This work was supported by the funding of Shanghai Municipal Health Commission (202240367), Capacity Building project of Local Colleges of Shanghai Science and Technology Commission (23010502800), and Shanghai Sailing Program (22YF1417900).

## Conflict of interest

The authors declare that the research was conducted in the absence of any commercial or financial relationships that could be construed as a potential conflict of interest.

## Publisher’s note

All claims expressed in this article are solely those of the authors and do not necessarily represent those of their affiliated organizations, or those of the publisher, the editors and the reviewers. Any product that may be evaluated in this article, or claim that may be made by its manufacturer, is not guaranteed or endorsed by the publisher.
