# Corrigendum: The neutrophil-to-lymphocyte ratio is associated with mild cognitive impairment in community-dwelling older women aged over 70 years: a population-based cross-sectional study

**DOI:** 10.3389/fnagi.2023.1297736

**Published:** 2023-10-11

**Authors:** Shengjie Li, Xiaoyu Chen, Mengze Gao, Xingyu Zhang, Peipei Han, Liou Cao, Jing Gao, Qiongying Tao, Jiayi Zhai, Dongyu Liang, Qi Guo

**Affiliations:** ^1^Department of Rehabilitation Medicine, Shanghai University of Medicine and Health Sciences Affiliated Zhoupu Hospital, Shanghai, China; ^2^School of Sports and Health, Tianjin University of Sport, Tianjin, China; ^3^Department of Nephrology, Molecular Cell Lab for Kidney Disease, Ren Ji Hospital, Shanghai Jiao Tong University School of Medicine, Shanghai, China; ^4^General Practice Clinic, Pujiang Community Health Service Center in Minhang District, Shanghai, China; ^5^Jiading Subdistrict Community Health Center, Shanghai, China; ^6^Clinical Research Center, Jiading District Central Hospital Affiliated Shanghai University of Medicine and Health Sciences, Shanghai, China

**Keywords:** inflammations, mild cognitive impairment, sex difference, population-based study, neutrophil-to-lymphocyte ratio (NLR)

In the published article, there was an error in the legend for Figure 2 Logistic regression of MCI and NLR after adjusted model in four subgroups by sex and age (A–D). as published. Because we are not familiar with the submitted page, we revised Figure 2 as suggested by the reviewers, figure 2 has made changes in word and PDF but has not been submitted to the system. The corrected legend appears below.

**Figure 2:**
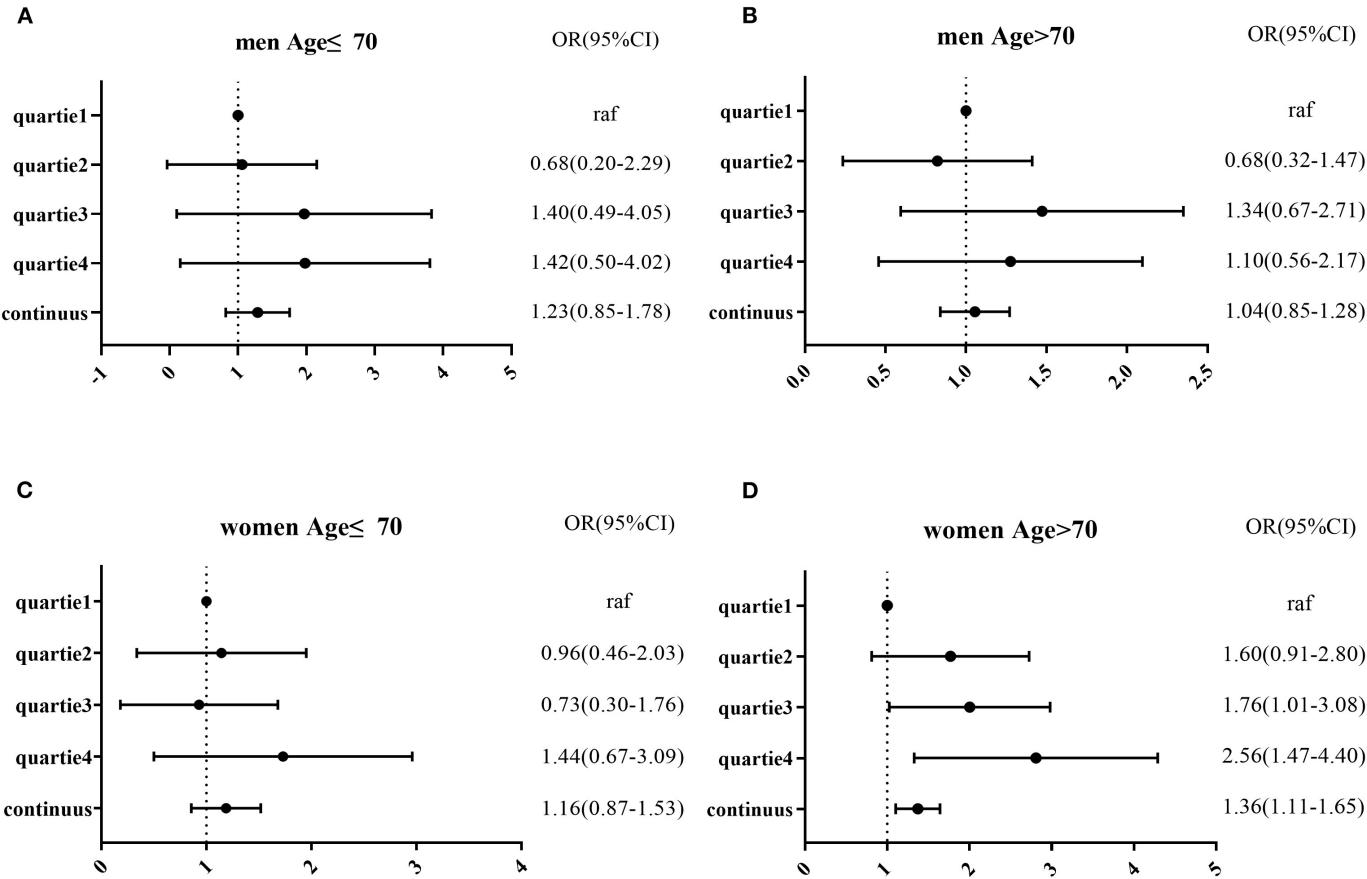
Logistic regression of MCI and NLR after adjusted model in four subgroups by sex and age **(A–D)**.

In the published article, there was an error. We revised the tables as suggested by the reviewers, but forgot to modify the content of the abstract and results.

A correction has been made to **Abstract**, “*Methods*”, 1. This sentence previously stated:

“A total of 3,126 individuals aged over 60 years in Shanghai were recruited for face-to-face interviews, and blood samples were collected.”

The corrected sentence appears below:

“A total of 3,169 individuals aged over 60 years in Shanghai were recruited for face-to-face interviews, and blood samples were collected.”

A correction has been made to **Abstract**, “*Results*”, 1. This sentence previously stated:

“MCI in women [odds ratio (OR) = 1.33; 95% confidence interval (CI) = 1.14–1.55]. In addition, the elevated NLR quartile was associated with an increased risk of MCI, especially in women older than 70 years (*p-*value for trend = 0.012).”

The corrected sentence appears below:

“MCI in women [odds ratio (OR) = 1.28; 95% confidence interval (CI) = 1.09–1.49]. In addition, the elevated NLR quartile was associated with an increased risk of MCI, especially in women older than 70 years (*p-*value for trend = 0.011).”

A correction has been made to **Results**, 3.

The sentence previously stated:

“Table 1 presents the characteristics of the study participants (*n* = 3,168) stratified by sex.”

The corrected sentence appears below:

“**Table 1** presents the characteristics of the study participants (*n* = 3,169) stratified by sex.”

The sentence previously stated:

“The prevalence of MCI was highest in the fourth quartile of the NLR [odds ratio (OR) = 2.10; 95% confidence interval (CI) = 1.35–2.35].”

The corrected sentence appears below:

“The prevalence of MCI was highest in the fourth quartile of the NLR [odds ratio (OR) = 2.10; 95% confidence interval (CI) = 1.35–3.25].”

The sentence previously stated:

“Table 3 shows that the prevalence of MCI was higher in the third (OR = 1.76; 95% CI = 1.01–3.05),”

The corrected sentence appears below:

“**Table 3** shows that the prevalence of MCI was higher in the third (OR = 1.76; 95% CI = 1.01–3.08),”

The authors apologize for this error and state that this does not change the scientific conclusions of the article in any way. The original article has been updated.

