# Supplementary material for: The neutrophil-to-lymphocyte ratio is associated with mild cognitive impairment in community-dwelling older women aged over 70 years: a population-based cross-sectional study
Source: Front Aging Neurosci. 2023 Sep 14;15:1261026. doi: 10.3389/fnagi.2023.1261026 (PMC10539551; doi:10.3389/fnagi.2023.1261026)
Supplement: Supplementary file 1 [file Table_1.pdf]

Supplementary Table. Baseline characteristics for all participants by sex group

Characteristic	Total (n=3169)	Men (n=1368)	Women (n=1801)	P value
Age (years)	72.18±5.34	72.53±5.36	71.91±5.31	0.001
BMI (kg/m^2)	23.85±3.36	23.85±3.20	23.86±3.49	0.913
Widow (%)	512 (16.2)	106 (7.7)	406 (22.5)	<0.001
Education (%)				<0.001
Illiteracy	311 (9.8)	48 (3.5)	263 (14.6)	
Primary school	1168 (36.9)	486 (35.5)	682 (37.9)	
Junior high school or above	1690 (53.3)	834 (61.0)	856 (47.5)	
Drinking (%)	735 (23.2)	574 (42.0)	161 (8.9)	<0.001
Smoking (%)	443 (14.0)	433 (31.70	10 (0.6)	<0.001
MNA	19.41±10.95	19.68±11.08	19.20±10.84	0.232
IPAQ (Met-min/wk)	4053 (1533-7413)	3465 (1418-6720)	4298 (1621-7812)	0.001
Chronic disease (%)				
Hyperlipidemia	1133 (35.8)	394 (28.8)	739 (41.0)	<0.001
Hypertension	2012 (63.5)	861 (62.9)	1151 (63.9)	0.574
Diabetes	640 (20.2)	287 (21.0)	353 (19.6)	0.338
Coronary heart disease	806 (25.4)	301 (22.0)	505 (28.0)	<0.001
Depression (%)	396 (12.5)	128 (9.40)	268 (14.9)	<0.001
MCI	324 (10.2)	116 (8.4)	208 (11.5)	0.005
Peripheral Blood Biomarkers				
WBC (×10^9/ L)	6.01±1.49	6.20±1.52	5.86±1.45	<0.001
Lymphocytes%	33.41±8.46	31.83±8.36	34.60±8.33	<0.001
Neutrophils%	59.61±9.44	60.58±9.58	58.87±9.27	<0.001
Lymphocytes (×10^9/L)	1.98±0.64	1.95±0.66	2.01±0.62	0.018
Neutrophils (×10^9/L)	3.61±1.18	3.78±1.20	3.48±1.15	<0.001
NLR	1.99±0.93	2.14±1.01	1.89±0.85	<0.001

BMI, body mass index; MNA, Mini-Nutritional Assessment; IPAQ, international physical activity questionnaire; MCI, mild cognitive impairment; WBC, white blood cell; NLR, neutrophil-to-lymphocyte ratio.
